# Expression of the muscle-associated gene *MYF6* in hairy cell leukemia

**DOI:** 10.1371/journal.pone.0227586

**Published:** 2020-02-10

**Authors:** Evgeny Arons, Hong Zhou, Mark Sokolsky, Daniel Gorelik, Katherine Potocka, Sarah Davies, Erin Fykes, Katherine Still, Daniel C. Edelman, Yonghong Wang, Paul S. Meltzer, Mark Raffeld, Adrian Wiestner, Liqiang Xi, Hao-Wei Wang, Maryalice Stetler-Stevenson, Constance Yuan, Robert J. Kreitman

**Affiliations:** 1 Laboratory of Molecular Biology, National Cancer Institute, National Institutes of Health, Bethesda, MD , United States of America; 2 Cancer Genetics Branch, National Cancer Institute, National Institutes of Health, Bethesda, MD, United States of America; 3 Laboratory of Pathology, National Cancer Institute, National Institutes of Health, Bethesda, MD, United States of America; 4 Laboratory of Lymphoid Malignancies, National Heart, Lung and Blood Institute, National Institutes of Health, Bethesda, MD, United States of America; University of Kentucky, UNITED STATES

## Abstract

Hairy cell leukemia (HCL) is a purine analog-responsive B-cell malignancy containing the BRAF V600E mutation, expressing CD22, CD11c, CD103, tartrate resistant acid phosphatase (TRAP) CD25, CD123, and annexin 1A. BRAF V600E and the latter 4 markers are usually absent in the more aggressive and chemoresistant variant HCLv. To evaluate differences between HCL and HCLv, expression microarrays comparing HCL with HCLv were performed for 24694 genes using 47323 probes. Microarray data from 35 HCL and 27 HCLv purified samples showed the greatest HCL-HCLv difference in the muscle-associated gene *MYF6*, expressed by its 2 probes 18.5- and 10.8-fold higher in HCL than HCLv (p<0.0001). By real-time quantitative PCR (RQ-PCR), 100% of 152 classic HCL samples were *MYF6*-positive, vs 5 (6%) of 90 blood donors. *MYF6*-expression was also detected in 18 (35%) of 51 with HCLv, 11 (92%) of 12 with HCL expressing unmutated IGHV4-34, 35 (73%) of 48 with chronic lymphocytic leukemia (CLL), and 1 (8%) of 12 with mantle cell lymphoma. Hypomethylation status of *MYF6* supported expression in HCL more than HCLv. Posttreatment blood samples becoming negative by flow cytometry remained *MYF6*+ by RQ-PCR in 42 (48%) of 87 HCL patients, and *MYF6* RQ-PCR could detect 1 HCL in 10^5^ normal cells. *MYF6*, universally expressed in HCL and in most CLL samples, may be a useful biomarker for these leukemias. Further studies are underway to determine the role of *MYF6* in HCL.

## Introduction

Classic hairy cell leukemia (HCL) is a B-cell malignancy with distinctive immunophenotype, typically having BRAF V600E mutation, and expressing CD20, CD22, CD25, CD11c, CD103, CD123, annexin A1 (Anxa1), and tartrate-resistant acid phosphatase (TRAP) [1–4]. Purine analogs achieve high rates of durable complete remissions (CR), often with minimal residual disease (MRD) [5, 6]. HCL variant (HCLv), recognized as a separate disorder [3, 7, 8], generally lacks CD25, CD123, annexin A1, TRAP, and BRAF V600E, responds more poorly to therapy, and survival from diagnosis is shorter [2, 9–12]. We reported that HCL expressing unmutated (>98% homology to germline) immunoglobulin heavy-chain variable (IGHV) rearrangement type IGHV4-34 expresses wild-type BRAF and has a poor prognosis like HCLv, whether immunophenotypically consistent with HCLv or HCL [12, 13]. Mutations within MAP2K1 encoding MEK1 have been found in HCLv and IGHV4-34+ HCL [14–16].

The human *MYF6* (human Myogenic Factor 6, *MRF4*, or Herculin) gene is mapped to 12q21 next to another myogenesis regulated factor, *MYF5* [17]. *MYF6* cDNAs were isolated first from human and mouse skeletal muscle, the only tissue in which expression of the corresponding mRNA was observed [18]. The MYF6 protein is a member of a family of trans-acting transcription factors, also known as myogenic regulatory factors, including MyoD1 (Myf3) [19], myogenin (MyoG, MYF4) [20, 21] , MYF5 and MYF6 [22]. Myogenic regulatory factors are involved in the development of skeletal muscle by controlling the expression of muscle specific genes [23]. Each of these four genes encodes a highly conserved basic-helix-loop-helix (bHLH) region that is responsible for the binding of Myf proteins to E-box sites (CANNTG) located in the promoter region of muscle-specific genes. They were reported expressed in normal tissue exclusively in striated muscle [18, 20, 22].

In neoplasia, *MYF6* expression was reported in 33% of rhabdomyosarcomas [23] and silent corticotroph macroadenomas [24]. *MYF6* gene hypomethylation was found in non-small cell lung cancer (NSCLC), associated with stage I disease [25]. Microarray-based expression of chronic lymphocytic leukemia (CLL) samples listed *MYF6* expression, associated with trisomy 12 [26], validated by real-time PCR [27]. To determine genes differentially expressed in HCL vs normal B-cells and other B-cell malignancies, samples from 10 HCL patients were compared with normal B-cells and samples from 46 patients with B-cell lymphomas or CLL, in microarray studies [28]. A total of 82 genes including *MYF6* were reported upregulated in HCL, and *MYF6* was one of 22 genes shown by immunohistochemistry to be expressed at the protein level [28]. Basso et al. reported *MYF6* expression among 8602 other genes in 336 samples which included 16 HCL [29]. To our knowledge *MYF6* expression in HCL was not further investigated, nor was it studied in HCLv. Using microarray studies, we decided to study genes upregulated in HCL as opposed to HCLv, initially without considering *MYF6*.

## Material and methods

### Patients and leukemic cells

Samples from patients with HCL, HCLv and other leukemias or controls were obtained via protocol 10-C-0066 approved by the National Cancer Institute Institutional Review Board. Patients gave written informed consent. All patients were adults and therefore did not require consent from parents or guardians. Per protocol, consent is waived in cases where patients died. Patients were consented from March 2010 to August 2019. Patients consented were either inquiring about clinical trials or had questions related to their disease and were invited to give consent to submit research samples. Demographic details of the patients are shown in Table 1. Patients included had a diagnosis of a hematologic malignancy, regardless of the status of their disease. The protocol also allowed recruitment of normal controls. Most of the samples were from patients with HCL and HCLv. The patient population was skewed toward those patients with multiply relapsed disease, although patients with newly diagnosed HCL were also consented. HCL and HCLv cells for microarray studies were obtained in sodium heparin tubes and purified by Ficoll centrifugation, followed by total B-cell isolation using the Dynabeads^™^ Untouched^™^ Human B Cells Kit (ThermoFisher Scientific). This procedure removes cells binding to CD2, CD14, CD16a, CD16b, CD36, CD43 and CD235a, including human T cells, monocytes, NK cells, macrophages, granulocytes, plasma cells, platelets, and erythrocytes (per the manual). The HCL and HCLv samples were >80% pure prior to microarray studies.

**Table 1:** Patients tested in microarray analysis.

	HCL	HCLv*	
N	35	27	
Age range (median)	29–75 (57)	42–87 (70)	p < 0.0001
Sex (M:F)	31:04:00	22:05	
Purine analog courses, range (median)	0–8 (1)	0–5 (1)	
Prior Splenectomy (% of patients)	31%	41%	
Prior Rituximab (% of patients)	40%	44%	
Unmutated IGHV4-34 (% of patients)	0%	41%	
BRAF inhibitor	0%	0%	
Leukemic cells/mm^3^, range (median)	29.4–134,000 (4928)	454–286,000 (17427)	p = 0.17

*For microarray analysis, the HCL group is IGHV4-34 negative, and the HCLv group includes IGHV4-34 positive (n = 11) and negative (n = 16) patients.

### Microarray RNA expression assay to compare HCL with HCLv

Total RNA was purified from cell pellets by the Qiagen AllPrep kit (Qiagen, Valencia, CA) following the manufacturer’s recommendations. Using the Ambion Illumina TotalPrep-96 RNA Amplification Kit (ThermoFisher Scientific, Waltham, MA), 100 ng total RNA was labeled and amplified. Use of oligo d(T) primer reverse-transcribed the RNA into cDNA from the 3-prime end. Subsequently, the cDNA underwent second strand synthesis and *in vitro* transcription to generate biotinylated cRNA. The labeled cRNA was hybridized to Illumina Human Ref-8 v3 Expression Bead Chips (Illumina, Inc., San Diego, CA). After washing, the Bead Chips were scanned using the Illumina Hi-Scan and images were processed and analyzed using Illumina Genome Studio v2011.1 software. All raw data were normalized with the R package Lumi using the function LumiN. Links between *MYF6* and other genes including *BRAF* were examined using Ingenuity® Variant Analysis^™^ software https://www.qiagenbioinformatics.com/products/ingenuity-variant-analysis from Qiagen, Inc.

### RQ-PCR for *MYF6*

Peripheral blood was collected using the PAXgene Blood RNA tubes (PreAnalytiX, Feldbachstrasse, Switzerland) and total RNA was extracted by the PAXgene Blood miRNA Kit (Qiagen), per manufacturer’s instruction. The 25 ul reaction mixture, containing 1–3 ug total RNA, 2 ul 10 mM dNTP mix (ThermoFisher Scientific) and 2 ul 0.5 ug/ml Oligo(dT)20 primer (ThermoFisher Scientific) was denatured at 65°C for 5 minutes and immediately chilled on ice. First strand cDNA synthesis was performed in a 20 ul reaction mixture also containing 8 ul 5x First Strand Buffer (ThermoFisher Scientific), 4 ul 0.1M DTT, 2 ul of 40 units/ul RnaseOUT and 0.5 ul of 200 U/ml SuperScriptTM III RnaseH- Reverse Transcriptase (ThermoFisher Scientific). The reaction was incubated at 50°C for 50 minutes, followed by 5 minutes at 80°C to inactivate Reverse Transcriptase, and then stored at –20°C. *MYF6* RQ-PCR was performed with a QuantStudio5 thermal cycler (Applied Biosystems, Beverly, MA). Briefly, cDNA was amplified in a 25 ul total volume per reaction using the *MYF6* TaqMan® Assay Hs01547104_g1 and the TaqMan GeneExpression Master Mix (ThermoScientific, Waltham, MA) per manufacture instructions. The reaction conditions were as follows: 50°C 2 min, 95°C 10 min followed by 40 cycles of 95°C for 15 sec and 60°C for 60 sec. The *MYF6* expression level was determined relative to *PGK1* gene expression level amplified using human *PGK1* HEX gene specific TaqMan assay (IDT, Coralville IA) using QuantStudio Software (Applied Biosystems, Beverly, MA). The sample was considered as negative when after 40 amplification cycles the amplification signal was not detected by the thermal cycle. By limiting dilution, *MYF6* RQ-PCR was able to detect 10 HCL and 20 CLL cells in 10^6^ normal cells.

### DNA methylation in HCL vs HCLv

Genome-wide DNA methylation profiling was performed on the HCL and HCLv samples using Illumina HumanMethylation450 BeadChips (Illumina) according to the manufacturer’s recommendations. Liquid handling occurred through use of a Tecan robot (Tecan Group LTD., Männedorf, Switzerland). The raw data file generated from the Illumina GenomeStudio was normalized with SWAN normalization implemented in the “lumi” R package. Two files were produced, one with the beta value for individual targets and another one with corresponding M values for the beta values. Final targets with significant cutoffs were filtered by first selecting for M values with FDR<0.05 (adjusted based on Benjamini-Hochberg procedure) unless otherwise indicated. Then absolute beta values with differences greater than 0.2 were chosen. Partek Genomics Suite, R packages of lumi, methlumi and other related R packages were used for data processing, analysis and data presentation. Expression and methylation data were uploaded to the Gene Expression Omnibus (GEO) database, NCBI tracking system #20434981

### Western blot for MYF6 protein

293 cells were transfected with pCMV6-*MYF6* (OriGene, Rockville, MD) using Attractene Transfection Reagent (Qiagen, Germantown, MD) and the total protein extracted by RIPA buffer (50 mM TrisHCl, pH 8.0, 150 mM NaCl, 5 mM EDTA, 1% NP-40, ThermoScientific, Waltham, MA). For Nuclear and cytosolic protein extraction, aliquots of 8.8x10^6^ patient cells were pelleted, then lysed with RIPA buffer at 4°C for 30 min with constant rocking. The nuclear and cytosolic proteins from the cell lines were then separated using a nuclear extraction kit (Active Motif, Carlsbad, CA) per manufacturer’s instructions. Protein content was then quantified using a DS-11 FX+ spectrophotometer (DeNovix, New Castle County, DE). For MYF6 detection, equal amounts of protein (30ug/sample) were loaded onto NuPAGE 14% Tris-Glycine gels (ThermoFisher, Waltham, MA). Nylon^+^ or PVDF membranes were stained with murine monoclonal antibody (Mab) SC-514379 followed by 115-035-072-anti-mouse-HRP (Jackson ImmunoResearch). Protein was detected using the K-12043-D10 Chemiluminescent Substrate kit (Advansta, San Jose, CA). Blots were then imaged using the Syngene Pxi gel documentation system (Syngene, Frederick, MD). GAPDH was detected on Nitrocellulose membranes using polyclonal antibody #9485 (Abcam, Cambridge, MA) followed by anti-mouse-HRP #115-035-166 (Jackson ImmunoResearch).

### Statistics

Statistical comparison of dichotomous variables was by Fisher’s exact. Comparison of 2 groups of continuous variables was by non-parametric Wilcoxon. Comparison of groups of patients for gene expression was performed by t-test, with corrected (stepped-up) p-values for multiple comparisons. P-values were 2-sided.

## Results

### Patient characteristics

As shown in Table 1, microarray comparison was performed for 35 HCL and 26 HCLv patients. Ten (39%) of the 26 HCLv patients had unmutated IGHV4-34+ HCLv. As expected, Table 1 shows that HCLv patients were older and had higher leukemic cells/mm^3^, although only the former comparison showed a significant difference. Table 2 shows clinical characteristics of the patients tested by RQ-PCR, including 154 patients with HCL, 51 with HCLv, and 12 with IGHV4-34+ HCLv. As expected, due to the higher tumor burden and poor response to treatment of HCLv, a higher percentage of patients with HCLv had prior splenectomy than patients with HCL (26 vs 6.5%, p = 0.0006). Prior splenectomy was also more common in IGHV4-34+ HCL than HCL (33% vs 6.5%, p = 0.011), consistent with the reported higher burden and chemoresistance in that variant [13].

**Table 2:** HCL/HCLv patients tested for Myf6 by RQ-PCR1.

N	154	51	12
Age range (median)	29–91 (53)	40–92 (69)	54–77 (65)
Sex (M:F)	125:29:00	40:11:00	11:01
Purine analog courses, range (median)	0–6 (1)	0–7 (1)	0–6 (2)
Prior Splenectomy (% of patients)	6.50%	26%	33%
Prior Rituximab (% of patients)	26.00%	29%	33%
BRAF inhibitor	0%	2%	0%
Leukemic cells/mm^3^, range	0.026–38849	30.6–286,000	13.2–110,000
Leukemic cells/mm^3^, median	38.6	3384	1217

### Expression of *MYF6* in HCL and HCLv by gene expression profile

**Fig 1:**
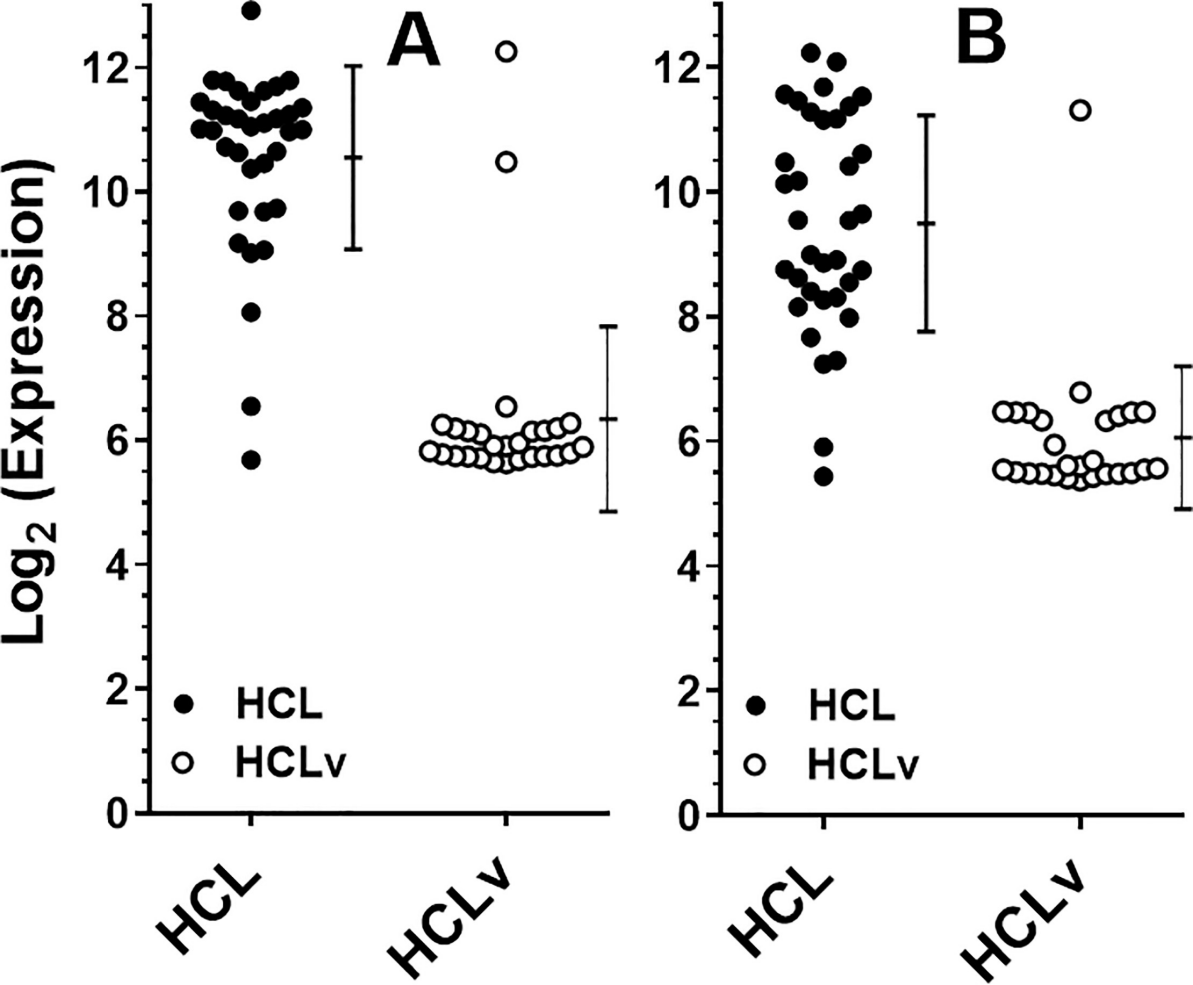
Microarray expression of Myf6 in HCL (●) and HCLv (○) assessed by probe 1805802 (A) and probe 2157717 (B). Error bars indicates standard deviations around the mean.

**Fig 2:**
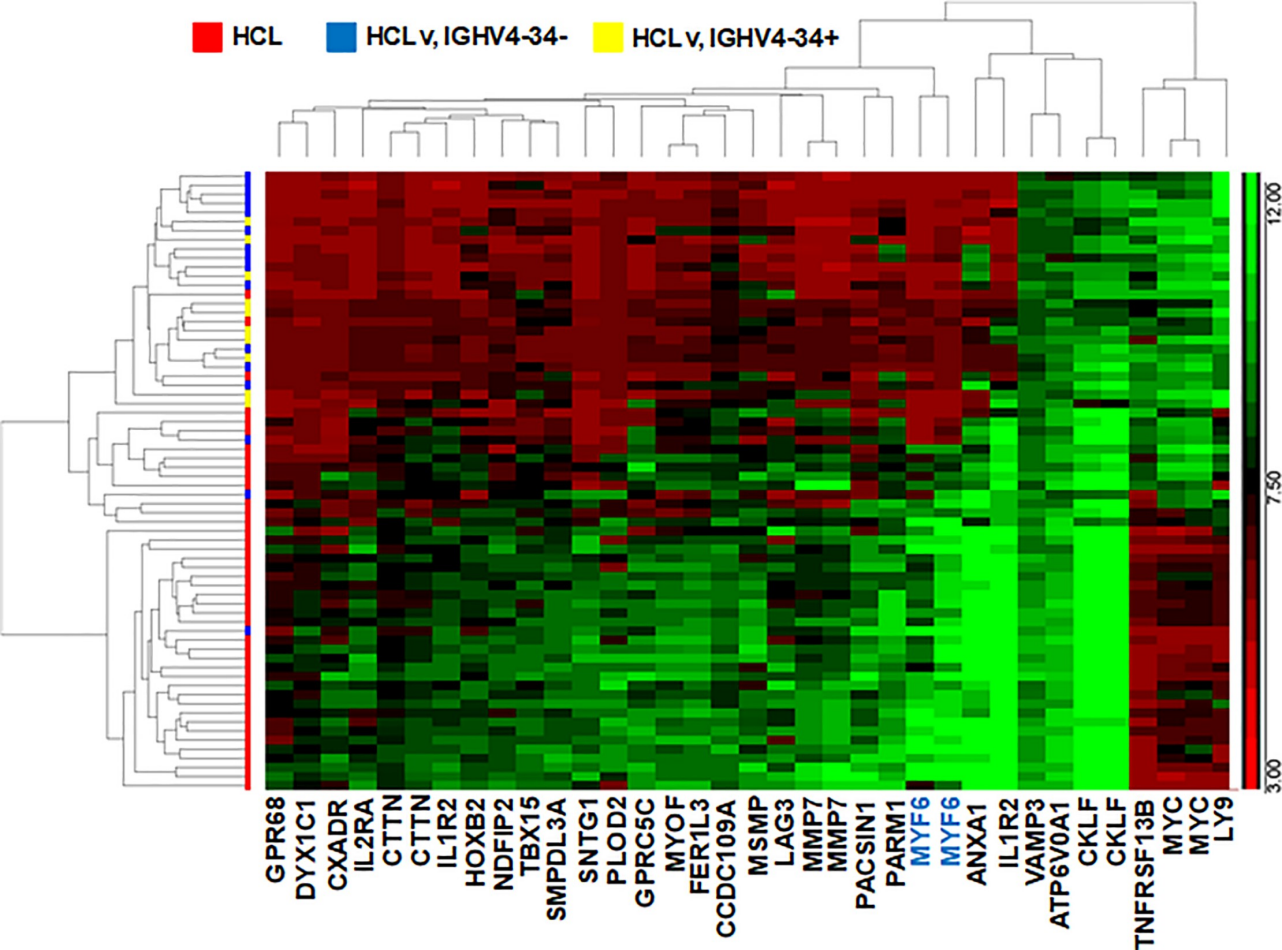
Heat map showing microarray results in HCL and HCLv. The 22 probes for 18 genes most different between HCL and HCLv are shown. The 2 groups compared included HCL and HCLv. HCLv patients with and without the IGHV4-34 IgH rearrangement are indicated in yellow and blue, respectively.

For microarray comparison of 35 patients with HCL and 27 with HCLv, leukemic cells were partially purified by CD11c sorting of the total B cell population obtained through negative B-cell selection of patient peripheral blood mononuclear cells (PBMCs). Microarray data from the 35 HCL and 27 HCLv patients showed that of 47323 probes for 24694 genes, the probe with the most difference, shown in Fig 1A and Table 3, was ILMN_1805802 for the muscle-related gene *MYF6*, with t-value 11.07 and stepped-up p-value 1.85 x 10^−11^. There was one other probe for *MYF6*, ILMN_2157717, which had the 13^th^ largest difference between HCL and HCLv, with a t-value of 8.89 and stepped-up p-value 5.11 x 10^−9^ (Table 3, Fig 1B). By these 2 probes, *MYF6* was expressed 18.5- and 10.8-fold higher in HCL than in HCLv (p<0.0001) in these 62 samples. A heat map showing the 33 probes for 30 genes with results most different between HCL and HCLv, including the 2 *MYF6* probes, is shown in Fig 2. *LY9*, *Myc*, and *TNFRSF13B* were expressed significantly more in HCLv than classic HCL, in contrast to *MYF6* and the other genes shown.

**Table 3:** Microarray data for probes with absolute T-score > 7.5 with mean log_2_ values.

Probe	Gene name	p-value	Stepped-Up p-value	t-score	HCL mean	HCLv mean
ILMN_1805802	MYF6	3.90E-16	1.85E-11	11.0741	10.5518	6.34454
ILMN_1812278	LY9	2.39E-14	5.66E-10	-9.9701	6.94974	10.4914
ILMN_1772131	IL1R2	3.63E-14	5.72E-10	9.86061	11.574	7.19092
ILMN_1810289	FER1L3	1.05E-13	1.24E-09	9.58132	8.87284	6.52241
ILMN_2393712	CTTN	2.44E-13	2.11E-09	9.36204	7.56989	6.40702
ILMN_1721580	TBX15	3.51E-13	2.11E-09	9.2675	8.3682	6.65334
ILMN_1810274	HOXB2	3.56E-13	2.11E-09	9.26373	8.12313	6.41951
ILMN_1744912	CTTN	5.15E-13	2.71E-09	9.16813	7.90934	6.15836
ILMN_3302919	MYOF	8.14E-13	3.85E-09	9.04963	8.65623	6.45097
ILMN_1727975	SNTG1	9.68E-13	4.17E-09	9.00494	8.54798	6.01691
ILMN_1685403	MMP7	1.35E-12	5.11E-09	8.9186	8.48796	6.00407
ILMN_2192072	MMP7	1.46E-12	5.11E-09	8.89878	8.82602	6.09417
ILMN_2157717	MYF6	1.51E-12	5.11E-09	8.89005	9.48963	6.06077
ILMN_1671142	GPR68	2.17E-12	6.83E-09	8.79753	7.28965	5.87043
ILMN_1677396	NDFIP2	2.82E-12	8.35E-09	8.72952	8.20379	6.56636
ILMN_2115135	MSMP	1.16E-11	3.23E-08	8.36718	8.83465	6.77264
ILMN_1796349	SMPDL3A	2.06E-11	5.42E-08	8.22071	8.46603	6.67415
ILMN_1751020	PACSIN1	2.42E-11	6.03E-08	8.1798	9.01672	6.32955
ILMN_1812523	DYX1C1	3.35E-11	7.92E-08	8.09713	7.4054	5.99661
ILMN_1813338	LAG3	3.99E-11	8.99E-08	8.05234	8.58416	6.39694
ILMN_1758371	IL1R2	5.67E-11	1.22E-07	7.96272	8.1564	6.38335
ILMN_2414027	CKLF	9.56E-11	1.97E-07	7.82987	11.8448	9.46051
ILMN_1656560	PARM1	1.11E-10	2.19E-07	7.79188	9.44199	6.99224
ILMN_1680618	MYC	1.38E-10	2.60E-07	-7.7375	7.22406	9.79519
ILMN_1683774	IL2RA	1.62E-10	2.94E-07	7.69639	8.06391	6.03332
ILMN_2184184	ANXA1	2.03E-10	3.41E-07	7.63872	11.0697	7.46993
ILMN_1672759	CCDC109A	2.07E-10	3.41E-07	7.6337	8.64863	7.24435
ILMN_1759075	TNFRSF13B	2.09E-10	3.41E-07	-7.6315	6.34244	8.68449
ILMN_1712389	CKLF	2.46E-10	3.88E-07	7.58977	12.1651	9.88559
ILMN_1752579	ATP6V0A1	2.75E-10	4.09E-07	7.56097	10.1704	8.8398
ILMN_2352090	GPRC5C	2.76E-10	4.09E-07	7.56024	8.71261	6.3762
ILMN_1796925	CXADR	3.09E-10	4.38E-07	7.53205	7.70429	5.98888
ILMN_1714527	VAMP3	3.22E-10	4.38E-07	7.52127	9.56498	8.64842
ILMN_1771599	PLOD2	3.27E-10	4.38E-07	7.51722	8.45477	6.23114
ILMN_2110908	MYC	3.33E-10	4.38E-07	-7.5126	6.9804	9.48558

### Expression of *MYF6* in classic HCL, HCLv and other B-cell malignancies by RealTime Quantitative PCR

To verify *MYF6* expression by HCL, *MYF6* cDNA was assessed in peripheral blood leukemic samples from 154 patients with classic HCL. As shown in Table 4, these included 147 patients with classic HCL known not to express IGHV4-34, and 7 with classic HCL not tested for IGHV4-34. In these 2 groups, 100% of the 154 patients were positive for *MYF6* by RQ-PCR. Rates of *MYF6* expression were lower for other hematologic malignancies, including 92% for IGHV4-34+ HCL, 32% for IGHV4-34-negative HCLv, 41% for IGHV4-34+ HCLv, 73% for CLL, 60% for marginal zone lymphoma (MZL) and 8% for mantle cell lymphoma (MCL). None of 20 patients with adult T-cell leukemia (ATL) expressed *MYF6*. Cells from 90 healthy donors were also tested, and 5 (6%) were positive. Thus, *MYF6* was positive by RQ-PCR in all patients with HCL, in most patients with CLL, MZL, and VH4-34+ HCL, and in a minority of patients with other hematologic malignancies or normal controls, including HCLv. Patients with unmutated IGHV4-34 were more likely *MYF6+* by RQ-PCR if classic HCL (92%) than HCLv (41%, p = 0.008).

**Table 4:** Myf6 RQ-PCR results.

Population	Total	Myf6-positive	Myf6-negative	p-value*	p-value**
HCL, non-IGHV4-34	147	147 (100%)	0		<0.0001
HCL, unknown VH	7	7 (100%)	0	1	<0.0001
HCLv, non-IGHV4-34	34	11 (32%)	23 (68%)	<0.0001	0.0003
HCLv, IGHV4-34	17	7 (41%)	10 (59%)	<0.0001	0.0004
HCL, IGHV4-34	12	11 (92%)	1 (8%)	0.076	<0.0001
CLL	48	35 (73%)	13 (27%)	<0.0001	<0.0001
HCL plus CLL	3	3 (100%)	0	1	0.0004
Normal donors	90	5 (6%)	85 (94%)	<0.0001	
ATL	20	0	20 (100%)	<0.0001	0.58
MCL	12	1 (8%)	11 (92%)	<0.0001	0.54
MZL	5	3 (60%)	2 (40%)	0.0009	0.0037

p-values by Fishers exact compared each group to HCL, non-IGHV4-34 (*) or to normal donors (**). Other comparisons included HCLv, non-IGHV4-34 vs HCLv, IGHV4-34 (p = 0.55), HCLv, IGHV4-34 vs HCL, IGHV4-34 (p = 0.0080), and HCLv, non-IGHV4-34 vs CLL (p = 0.0003).

### DNA methylation in *MYF6* gene region

**Fig 3:**
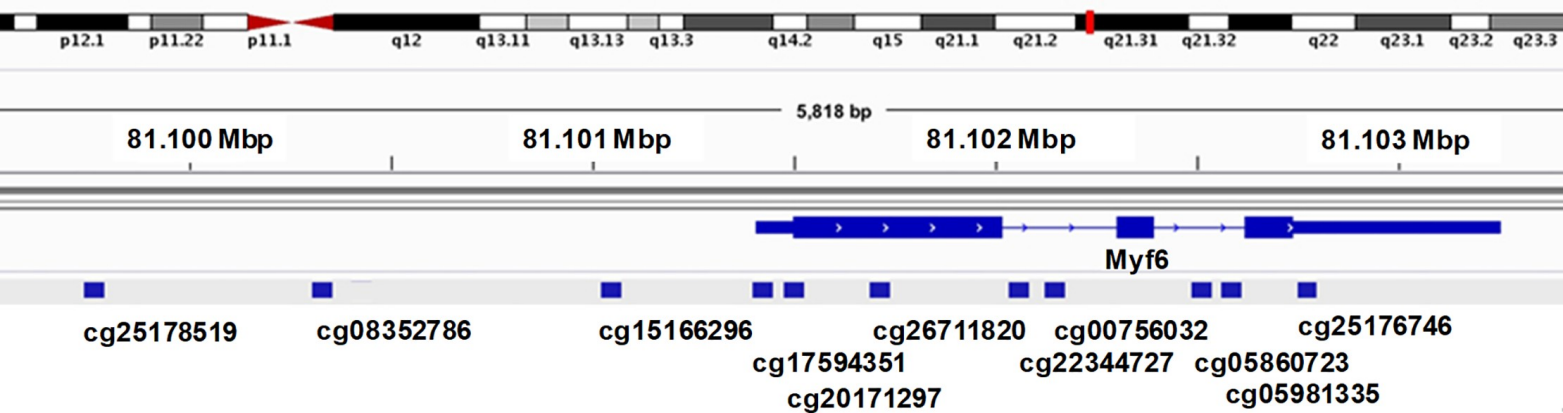
Locations for Myf6 methylation probe binding. Blue squares showing the binding site are situated over the ‘cg’ label of each probe.

Since methylated cytosines in the context of cytosine guanine dinucleotides (CpGs) cluster at high density in regions of DNA termed CpG islands which associate with gene promoters [30], and methylation of promoter-associated CpGs is associated with transcriptional repression and gene silencing [31], we determined whether the gene for *MYF6* is hypomethylated more often in HCL than in HCLv. We conducted genome-wide DNA methylation profiling using Illumina HumanMethylation450 BeadChips and 476,882 probes. We compared 34 classic HCL patients to 28 with HCLv, the HCLv group including IGHV4-34+ (n = 13) and IGHV4-34-negative (n = 15). As shown in Table 5, 8 out of 11 *MYF6* cg probes had significant difference in HCL vs HCLv with respect to stepped up p-value <0.05. The 280^th^ most different probe, cg05981335, had a t-value of -7.142, and stepped-up p-value 2.44 x 10^−6^. Only 1 of these 8 probes had a positive t-value indicating more hypomethylation in HCLv than HCL. This probe binds to the reverse strand and its significance is unknown. Of the remaining probes, cg08352786, cg17594351, cg15166296, and cg25178519, based on the DBTSS database (https://dbtss.hgc.jp/), bind within -2.5 kb and +1.0 kb of an annotated transcription start site (TSS) and would be considered as binding within the promotor region (Fig 3). Thus, the DNA methylation data supported more hypomethylation of *MYF6* in HCL than HCLv, consistent with the higher level of expression in HCL than HCLv.

**Table 5:** Myf6 probes showing differences in methylation between HCL and HCLv.

Probe	Place	P-value	Stepped-up p-value	t-value	Strand	UCSC RefGene Group	Relation to UCSC CpG Island
cg05981335	280	1.43E-09	2.44E-06	-7.142	F	Body	Island
cg05860723	295	1.82E-09	2.93E-06	-7.082	R	Body	Island
cg25176746	663	7.42E-08	5.34E-05	-6.131	F	3'UTR	S_Shore
cg22344727	741	1.17E-07	7.52E-05	-6.013	R	Body	Island
cg08352786	828	1.75E-07	0.000101	5.908	R	TSS1500	N_Shore
cg17594351	2890	1.58E-05	0.002613	-4.697	F	TSS200	N_Shore
cg00756032	5134	0.0001	0.009465	-4.163	R	Body	Island
cg20171297	6967	0.00025	0.017133	-3.894	R	1stExon;5'UTR	N_Shore
cg15166296	18410	0.00308	0.079676	-3.085	F	TSS1500	N_Shore
cg26711820	20008	0.00371	0.088353	-3.02	F	1stExon	N_Shore
cg25178519	36755	0.0141	0.182887	2.529	R	TSS1500	N_Shore

### Presence of *MYF6* protein in HCL cells

**Fig 4:**
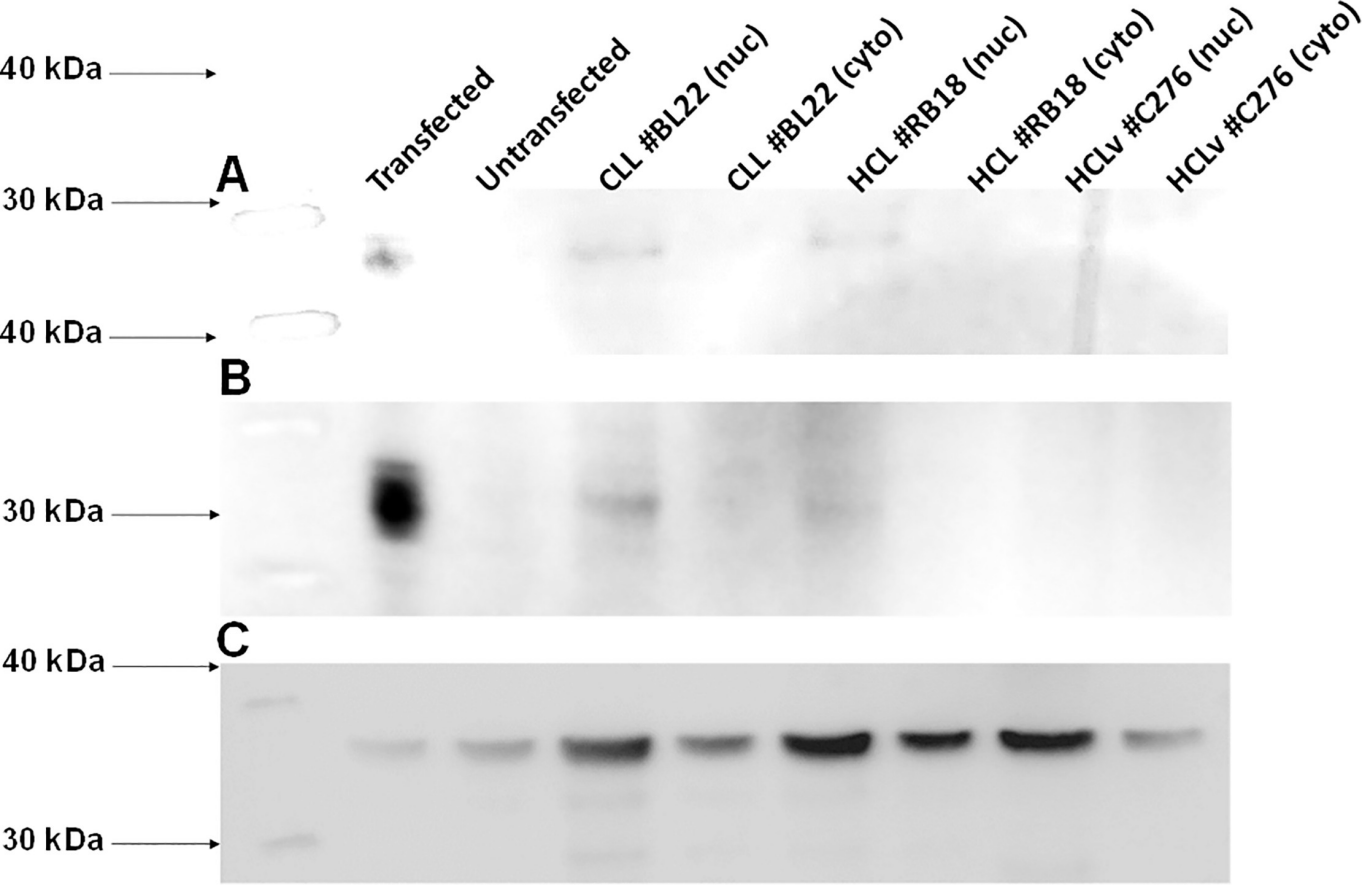
Western blot for Myf6 protein in HCL and CLL cells. Nylon^+^ (A) and PVDF (B) membranes were stained with murine Mab SC-514379 followed by anti-mouse-HRP. In C, PVDF was stained with polyclonal anti-GAPDH antibody followed by anti-mouse-HRP. Lanes include Myf6-transfected (lane 1) and untransfected (lane 2) 293 cells, nuclear and cytoplasmic fractions for CLL patient BL22 (lanes 3 & 4, respectively), HCL patient BL18 (lanes 5 & 6), and HCLv patient C276 (lanes 7 & 8). In each lane, 30 ug of total protein was added, except less in A lane 1 to obtain bands of similar intensity.

To investigate whether the high mRNA levels of MYF6 correspond to expression of MYF6 protein, we tested for the presence of MYF6 protein on HCL and HCLv cells obtained from patients, using anti-MYF6 Mab. We expected that since MYF6 is a transcription factor, its expression would be difficult to detect by western blot. In fact, other than transfected 293 cells (Fig 4, lane 1), we could not detect any MYF6 protein except for faint bands in the nuclear fraction of MYF6+ cells. The 2 patients shown with CLL and HCL were also positive by RQ-PCR and the HCL patient was also MYF6+ by microarray. As expected, we could not detect significant levels of MYF6 in the nuclear fraction of the patient with HCLv who was negative for MYF6 by RQ-PCR and microarray. The lower expression of GAPDH in lanes 1 and 2 is consistent with much higher total protein expression by 293 cells compared to primary leukemia cells, and thus a lower percentage of GAPDH in a 30 ug gel sample. Similarly, GAPDH from the cytosolic fraction of primary cells from the HCLv patient (lane 8), who had rapid disease progression, possibly constituted a lower percentage of the 30 ug of total protein loaded. We conclude that HCL cells not only express *MYF6* mRNA but also its protein product.

### Determination of limits of detection of MRD by *MYF6*

To determine the sensitivity of *MYF6* detection by RQ-PCR, we diluted *MYF6*+ HCL and CLL cells into normal peripheral blood mononuclear cells and determined the greatest dilution which could still be detected in most biologic replicates. We could reliably detect HCL at an approximate dilution of 1 in 10^5^ normal cells, with 3 of 6 and 3 of 4 replicates positive at 3/10^6^ and 10/10^6^, respectively (Table 6). We could detect CLL at an approximate dilution of 2 in 10^5^ normal cells, with 1 of 6 and 4 of 6 replicates positive at 10/10^6^ and 20/10^6^, respectively. Cycle threshold (C_T_) values are shown. Thus, in some patients with CLL and HCL, *MYF6* RQ-PCR can be used to determine MRD. Of 87 HCL patients with repeat post-treatment blood samples negative for HCL by flow cytometry, 42 (48%) were still positive for *MYF6*. Conversely, of 76 patients with repeat post-treatment blood samples negative for *MYF6* by RQ-PCR, 13 (17%) were positive by flow cytometry, at a median of 0.3 HCL cell/mm^3^. Thus, HCL cells from some patients may have lower expression of *MYF6* and require more cells than others for positivity, while some patients with higher expression could be followed by *MYF6* RQ-PCR more easily and sensitively than flow cytometry. Since Myf6 RQ-PCR could be more sensitive or less sensitive than flow cytometry in different patients, we could not report sensitivity and specificity of this assay in patients after treatment. Since 6% of normal donors were *MYF6*+, some patients, including those with *MYF6*+ monoclonal B-cell lymphocytosis (MBL), may not become *MYF6* negative after resolution of circulating HCL. Additional testing will be needed to better define the sensitivity and specificity of *MYF6* RQ-PCR relative to flow cytometry, or other methods including RQ-PCR using patient CDR3-specific primers [32] or deep sequencing [33].

**Table 6:** Sensitivity of Myf6 detection in CLL and HCL.

Dilution HCL patient:	1/10^6^	3/10^6^	10/10^6^	20/10^6^	50/10^6^	10^2^/10^6^	10^3^/10^6^
Number positive	2 of 6	3 of 6	3 of 4	3 of 4	4 of 4	4 of 4	2 of 2
PRGK1 C_T_ (median of +’s)	26.5	26.4	27.2	27	26.2	26.5	26.5
Myf6 C_T_ (median of +’s)	36	37	35.8	37	36.4	34	31
PRGK1-Myf6 ΔC_T_	9.5	10.6	8.5	10.1	10.3	7.5	4.5
Dilution CLL patient							
Number positive			1 of 6	4 of 6		5 of 6	2 of 2
PRGK1 C_T_ (median of +’s)			29.4	26.9		26.1	25.8
Myf6 C_T_ (median of +’s)			39.9	38.7		36.9	32.8
PRGK1-Myf6 ΔC_T_			10.5	11.8		10.8	6.9

Cycle threshold (C_T_) values are presented to the nearest tenth of a cycle. ΔC_T_ values, the differences between C_T_ values for the housekeeping gene PRGK1 and Myf6, were calculated using C_T_ values with several decimal places.

## Discussion

MYF6 is a muscle specific transcription factor normally expressed in skeletal muscle, but not other types of normal tissues. *MYF6* expression has been reported for a narrow group of solid tumors including of rhabdomyosarcoma [23] and corticotroph macroadenomas [24]. Although data from microarray studies by Basso et al. in HCL was listed [28, 29], its detailed expression in HCL and HCLv was not reported. To determine genes differentially expressed in HCL as opposed to HCLv, we performed expression microarray studies for 24694 genes. We were surprised to find that a probe for *MYF6* had the lowest p-value for preferential expression in HCL compared to HCLv. We found that 100% of 154 HCL samples were positive for *MYF6* by RQ-PCR, with less percentages of other hematologic malignancies positive. Additionally, we discovered that most CLL samples were positive for *MYF6*. Increased expression of *MYF6* correlated with overall hypomethylation in (CpGs)CpG probes with HCL vs HCLv. MYF6 protein could be detected in the nuclear fraction of *MYF6*+ HCL and CLL cells, and in these leukemias, *MYF6* PQ-PCR could be used for MRD detection with sensitivities of 1/10^5^ and 2/10^5^, respectively.

### Expression of *MYF6* in HCL and other B-cell malignancies from Oncomine and Gene Expression Omnibus

The *MYF6* expression data for classic HCL was available from normal and transformed array expression profiles GSE2350 contributed by Basso et al. [28, 29]. Their microarray assay studied 8603 genes in 336 normal and malignant B-cell samples, including from 10 patients with HCL. This study identified 82 genes upregulated in HCL including *MYF6*, and MYF6 was one of 22 proteins detectable on HCL by IHC [28]. This data set includes a median *MYF6* expression level of 3.75 for 16 patients with HCL, compared to -0.6 to -1.9 for Burkitt’s Lymphoma (n = 127), centroblastic lymphoma (n = 28], CLL (n = 34], diffuse large B-cell lymphoma (n = 41], follicular lymphoma (n = 6), Hodgkin’s lymphoma (n = 4), MCL (n = 8), plasma cell leukemia (n = 3) and primary effusion lymphoma (n = 9). Median expression levels were -0.5 to -1.2 for 5 samples each of B-lymphocytes, centroblasts, memory B-cells, naïve pregerminal center B-cells, and small cleaved follicular center cells [29]. Thus, *MYF6* is an important gene expressed in HCL compared to either HCLv or B-cells from malignant or benign sources. It is interesting that this reported data set shows *MYF6* expression much higher in HCL (3.75, n = 16) than in CLL (-0.65, n = 34), since we found most CLL patient samples *MYF6*+. However, while *MYF6* expression as reported by Basso et al. [28, 29] was lower in CLL than in HCL, CLL was higher than nearly all other non-HCL malignancies, and this is consistent with our data showing that 73% of CLL patients were *MYF6*+ by RQ-PCR. *Myf6*, which is expressed on chromosome 12, was reported by Porpaczy et al., to be preferentially expressed in 29 trisomy-12 CLL patients vs 32 non-trisomy-12 patients [27].

### *MYF6* expression by normal cells

We found that 5 of 90 normal donors were *MYF6*+ by RQ-PCR. Since we found that *MYF6* in CLL can be detected at 2 cells per 10^5^, it is possible that some of the normal donors may have had low levels of monoclonal B-cells. It has been reported that monoclonal B-lymphocytes can be detected in ~5% of adults over the age of 40 [34], which probably explains the *Myf6* positivity of 6% of uncharacterized normal donors. Myf6 would not be an accurate target for MRD in HCL patients with MBL, but the presence of MBL is easily detected by flow cytometry prior to treatment, and the remaining ~95% of HCL patients would be evaluable. Normal blood donors were anonymous and flow cytometry was not performed on each sample. We found no *MYF6* expression in malignant T-cells (Table 4). Our data showing that samples from 94% of normal donors are Myf6 negative seem inconsistent with data from Porpaczy et al. that 6 of 6 normal donors had CD19+ cells which were low+ for Myf6 [27]. The reason for the discrepancy may relate to the fact that RQ-PCR on our 90 normal donors, like our 305 leukemic patients, was performed on RNA purified from PaxGene tubes of whole blood; it is possible that RQ-PCR on CD19-selected B-cells could give different results.

### Expression of *MYF6* in IGHV4-34+ HCL, and investigation of the function of Myf6

We reported that patients with unmutated IGHV4-34+ HCL, indistinguishable from classic HCL immunophenotypically, have clinical features more like HCLv than HCL, including poor response to single-agent purine analog [13]. This molecularly defined variant was reported as negative for BRAF V600E [12, 35]. We found in this study that *MYF6* was positive in a higher percentage of IGHV4-34+ HCL patients than patients with HCLv, 11 of 12 vs 18 of 51 (p = 0.0007). Yet it was not associated with IGHV4-34 in particular, since similar rates of Myf6 positivity were observed in IGHV4-34 positive (41% of 17) and negative (32% of 34) HCLv (Table 4, p = 0.55). We consider patients HCL rather than HCLv if their CD25 is + or bright positive, regardless of BRAF status, since the latter is not universally measured, while patients wild-type for BRAF and negative or dim+ for CD25 are considered as HCLv. To determine a possible link between *MYF6* and either BRAF V600E or other MAPK pathways involved in HCL pathogenesis, we interrogated the Ingenuity software application for all proteins possibly interacting with Myf6, and did not find interactions with any known proteins of the MAPK pathway. This together with our experiments indicates that the expression of *MYF6* is not dependent on the BRAF V600E mutation and may be related to other factors including CD25 expression. While the function of *Myf6* in HCL is unknown, it is unlikely to have a causative role since it is observed in some other hematologic malignancies like CLL. We determined using the Cancer Cell Line Encyclopedia (https://portals.broadinstitute.org/ccle) whether Myf6 expression correlates to markers which are selective for HCL compared to HCLv. Using 191 hematologic cell lines, there was no correlation between Myf6 and either CD25, CD134, TRAP, or Annexin 1a. There are no cell lines resembling classic HCL to the extent that they express BRAF V600E, although there are several resembling HCLv [36, 37]. None of the 191 hematologic cell lines examined were HCL or HCLv-like.

Additional work will be required to address the question of why *MYF6*, a muscle specific transcription factor, is expressed by all classic HCL. *MYF6* involvement in pathways specific for HCL but not most cases of HCLv would suggest an important pathogenesis effect. Efforts to produce *MYF6* knockout cells are among studies underway to investigate this hypothesis.

## Supporting information

S1 Fig Uncut gels for Fig 4A, 4B and 4C are shown. (PPTX)
