# Supplementary figures and images for: Expression of the muscle-associated gene MYF6 in hairy cell leukemia
Source: PLoS One. 2020 Feb 10;15(2):e0227586. doi: 10.1371/journal.pone.0227586 (PMC7010284; doi:10.1371/journal.pone.0227586)

## Slide 1
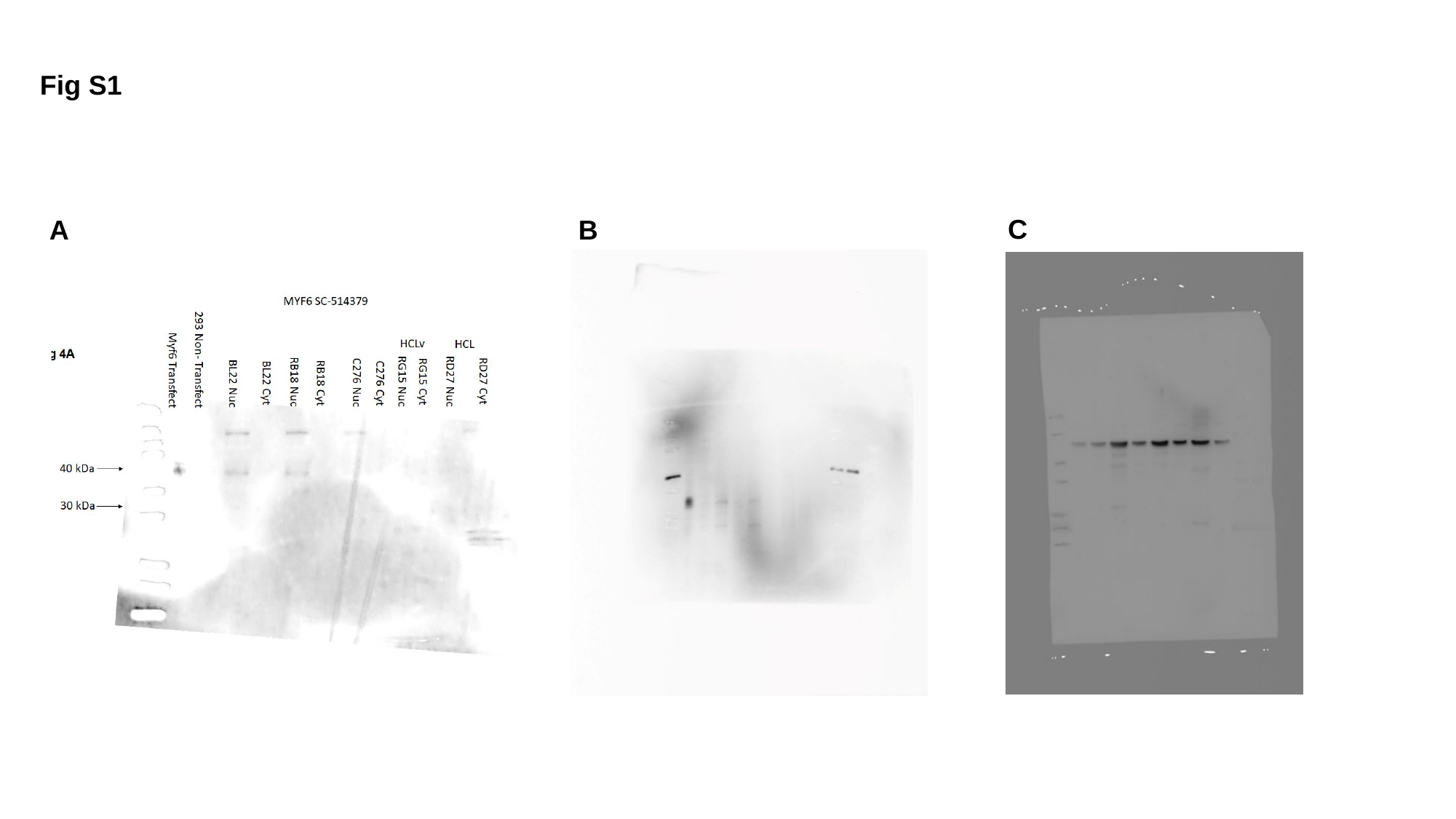

Fig S1
C
A
B

Supplement: S1 Fig — (PPTX) [file pone.0227586.s001.pptx]
